# Unravelling the Biological Potential of *Pinus pinaster* Bark Extracts

**DOI:** 10.3390/antiox9040334

**Published:** 2020-04-20

**Authors:** Pedro Ferreira-Santos, Zlatina Genisheva, Cláudia Botelho, Joana Santos, Carla Ramos, José A. Teixeira, Cristina M.R. Rocha

**Affiliations:** 1 CEB—Centre of Biological Engineering, University of Minho, Campus de Gualtar, 4710-057 Braga, Portugal; 2 CISAS—Centro de Investigação e Desenvolvimento em Sistemas Agroalimentares e Sustentabilidade, Escola Superior de Tecnologia e Gestão, Instituto Politécnico de Viana do Castelo, Rua Escola Industrial e Comercial de Nun’Álvares, 4900-347 Viana do Castelo, Portugal

**Keywords:** *Pinus pinaster* bark, extraction, pine bark extracts, phenolic compounds, flavonoid compounds, antioxidant activity, antihyperglycemic activity, antimicrobial activity, cells’ metabolism

## Abstract

Natural compounds from agro-food by-products have fostered interest in food industries. The aim of this study was to unravel potential uses for *Pinus pinaster* bark extracts (PBE). As functional features of this type of extracts are usually attributed to phenolic compounds, the extraction process was studied. Different PBEs were achieved, with high content in phenolic compounds, using different water/ethanol combinations as a solvent. These PBEs were chemically characterized, and their bioactivity and in vitro cell viability were evaluated. Extracts obtained with hydroethanolic solvents had higher content in phenolic and flavonoid compounds. All the PBEs presented high antioxidant, antibacterial and antihyperglycemic activities. Moreover, PBEs have low cytotoxicity and a selective activity against cancer cells as these were negatively affected. These features may allow the extracts to be used in food formulation and processing (as preservatives, antioxidants or bioactive ingredients), but they showed also potential for the pharmaceutical or nutraceutical sectors.

## 1. Introduction

Natural compounds, such as phenolics, flavonoids, proteins, carotenoids, among others, have fostered interest in different industries including paints, fertilizers, surfactants, textiles, rubbers, pharmaceuticals, etc. [1]. Additionally, in food industry technology, they are used as natural preservatives against oxidation and microorganisms (bacterial and fungal contaminations), and in the development of functional food ingredients [2].

The biological activity of plant extracts can be attributed to secondary metabolites such as phenolic acids, flavonoids and other phenolic compounds. The popularity of these extracts is linked to their biological properties, such as antioxidant, anti-inflammatory, antimicrobial, antiviral, antiatherogenic, etc. [3,4,5,6,7]. In particular, the extracts of *Pinus pinaster* (a conifer plant found in some Mediterranean countries and used in afforestation of Africa, New Zealand and Australia), are rich in phenolic acids, flavanols and flavonoids (e.g., cinnamic acid, hydroxybenzoic acid, catechin, quercetin and taxifolin) with a potent antioxidant activity [5,8]. These extracts have demonstrated beneficial effects for the treatment of several diseases, such as cardiovascular, metabolic, neurological, etc. [8,9,10,11]. The activities reported for *P. pinaster* extracts make this underexploited by-product (bark) of the wood industry of high interest for the pharmaceutical and food industries. There are only a few studies about the potential bioactivities and toxicity of the extracts from *P. pinaster* bark. However, the existent studies are focused on the commercial product Picnogenol^®^, an aqueous extract used as an active supplement [10,11].

The extracts from *P. pinaster* bark are predominantly obtained by conventional solvent extraction, Soxhlet, microwaves, supercritical CO_2_ and, more recently, by ohmic heating assisted extraction [5,12,13,14,15]. However, the variety of extraction conditions (such as type of solvent, solid-liquid ratio, time and temperature) may potentially affect the processes’ yield and the phenolic profile of the extracts. To prevent the environmental impact and reduce the bio-wastes and by-products of the agro-food industry, it is necessary to optimize the recovery of bioactive compounds with high added value and to enable their re-introduction in the market [16,17,18]. In addition, the applied methodology needs to be sustainable and “green” by using alternative and highly clean and nontoxic solvents [19].

It is known that polar solvents, such as ethanol or aqueous mixtures containing ethanol, are frequently used for the recovery of phenolic compounds from plant tissues [16]. In this sense, the aim of this research work was to study the functional potential of phenolic-rich extracts from pine bark (PB) using water and water-ethanol in different ratios as a solvent. The pine bark extracts (PBE) with the highest phenolic compounds’ content and highest in vitro antioxidant activity were chemically characterized, and its bioactivity (antioxidant, antimicrobial and antidiabetic) and in vitro cell viability (in normal and cancer cell lines) were evaluated.

## 2. Materials and Methods

### 2.1. Chemicals

Folin-Ciocalteu reagent, 2,2′-Azino-bis(3-ethylbenzothiazoline-6-sulfonic acid) diammonium salt (ABTS), 2,2-Di(4-tert-octylphenyl)-1-picrylhydrazyl (DPPH), 2,4,6-Tris(2-pyridyl)-s-triazine (TPTZ), 6-hydroxy-2,5,7,8-tetramethylchroman-2-carboxylic acid (Trolox), porcine pancreatic α-amylase (EC 3.2.1.1, type VI), *Saccharomyces cerevisiae* α-Glucosidase (EC 3.2.1.20, type I), *p*-nitrophenyl-R-d-glucopyranoside (pNPG), aluminium chloride (AlCl_3_), acarbose, Dulbecco’s Modified Eagle Medium (DMEM), fetal bovine serum (FBS), penicillin-streptomycin solution, resazurin sodium salt, dimethyl sulfoxide (DMSO, ≥99.9%) and all standard markers for HPLC were obtained from Sigma-Aldrich (St. Louis, MO, USA). Other reagents were analytical grade, and ultra-pure water was used throughout the experiments.

### 2.2. Raw Material Preparation and Characterization

Bark from *P. pinaster* (approximate age 15 years) was collected in Ponte de Lima, Portugal (April 2016). Firstly, the bark was washed with distilled water and dried at 40 °C for 48 h and subsequently milled in a cutting mill (Retsch SM 2000) to a granulometry of 0.1–0.45 mm for general chemical composition and 1–1.6 mm for extraction process.

Chemical summative analyses were determined in accordance to the National Renewable Energy Laboratory (NREL) official protocols, and included ethanol extractives (NREL/TP-510-42619), structural carbohydrates (namely cellulose and hemicellulose), klason and acid soluble lignin (NREL/TP-510-42618) and ash content (NREL/TP-510-42622). The mineral content was determined by inductively coupled plasma atomic emission spectrometry (ICP-AES), after PB digestion with HNO_3_. Fat content was determined according to the official AOAC method (nº 920.39). Total proteins content estimated by using the N×6.25 conversion factor, was performed using a Kjeldahl distillator (Kjeltec 8400 Analyzer, FOSS, Hilleroed, Denmark) by quantification of Nitrogen after PB digestion. PB moisture was determined gravimetrically using a moisture analyzer (MAC 50/1/NH, RADWAG, Radom, Poland). All experiments were performed in triplicate.

### 2.3. Extraction Conditions and Extracts Preparation

In the first part of the work, the procedure to study the influence of the extraction parameters of phenolic compounds present in PB was carried out using 100 mL cylindrical reactors duly protected from light in thermostatized water bath with shaking (170 rpm). The volume of extraction for all experiments was 40 mL. Experiments were performed using response surface methodogy RSM (2^3^ central composite design) for solid-liquid extraction with water or hydroethanolic solvent (30–90% (*v*/*v*)). The levels of independent variables were selected based on the results obtained from our preliminary experiments (data not shown) and data from the literature [14,15]. The five levels of each of the three variables were coded in 18 runs (including four replicates of the center point) and were performed in a random order. Independent variables for extraction were time (min, *x_1_*), temperature (°C, *x_2_*) and solid: liquid ratio (g/mL, *x_3_*). Dependent variables (*Y_1_* and *Y_2_*) were total phenolic content (TPC, mg GAE/g PB) and ferric reducing antioxidant power (FRAP, mmol Fe^2+^/g PB), respectively. Coded and actual values of the independent variables together with data of dependent variables are given in Table 1. Data were correlated following the polynomial Equation (1).

(1) $$Y_{i}=\beta_{0i}+\beta_{1i}x_{1}+\beta_{2i}x_{2}+\beta_{3i}x_{3}+\beta_{11i}x_{1}^{2}+\beta_{22i}x_{2}^{2}+\beta_{33i}x_{3}^{2}+\beta_{12i}x_{1}x_{2}+\beta_{13i}x_{1}x_{3}+\beta_{23i}x_{2}x_{3}$$

where, *Y_i_* correspond to the dependent variables; *x_1_, x_2_* and *x_3_* value of independent variables; *β_0i_, β_1i_, β_2i_, β_3i_, β_11i_, β_22i_, β_33i_, β_12i_, β_13i_ and β_23i_* are regression coefficients calculated from experimental data by multiple regression using the least-squares method.

The experimental data were fitted to the proposed model using *Statistica* software (Statistica 8.0). The statistical analysis was performed using ANOVA, which established the model significance, the significance for each polynomial coefficient, and the determination coefficient *R*^2^.

For the best conditions selected in the previous stage, new experimental assays were carried out in order to characterize and evaluate the bioactive potential and cell viability of extracts. The obtained extracts were dried by freeze drying and keep at 4 °C for further analysis.

### 2.4. Chemical Analysis of Extracts

#### 2.4.1. Total Phenolic Content (TPC)

The total content of phenolic compounds was measured by the Folin–Ciocalteu method that was based on the colorimetric reduction/oxidation reaction of phenols [5,20]. Gallic acid was used to perform the standard curve (*R*^2^ = 0.996) and the results were expressed as milligram gallic acid equivalents (GAE) per gram of pine bark (optimization process) or dry extract (extract characterization).

#### 2.4.2. Total Flavonoid Content (TFC)

The applied method for the determination of total flavonoids content has been previously described by Barros et al. [21]. An aliquot (500 µL) of the PBE solution was mixed with distilled water and NaNO_2_ solution (5%). After 6 min, AlCl_3_ solution (10%) was added and allowed to stand further 6 min; thereafter, NaOH solution (4%) was added to the mixture. Then, the mixture was properly mixed and allowed to stand for 15 min, and the absorbance was measured at 510 nm. (+)-Catechin was used to calculate the standard curve (*R*^2^ = 0.997) and the results were expressed as mg of catechin equivalents (CE) per g of extract (mg CE/g extract).

#### 2.4.3. UPLC Chromatography

Identification and quantification analysis of phenolic presents in PBE were performed as described previously [5] using a Shimatzu Nexpera X2 UPLC chromatograph equipped with Diode Array Detector (DAD) (Shimadzu, SPD-M20A, Columbia, MA, USA). Separation was performed on a reversed-phase Aquity UPLC BEH C18 column (2.1 mm × 100 mm, 1.7 µm particle size; from Waters, Milford, MA, USA) and a pre-column of the same material at 40 °C. The HPLC grade solvents used were water/formic acid (0.1%) and acetonitrile as eluents and the flow rate was 0.4 mL/min. Phenolic compounds were identified by comparing their UV spectra and retention times with that of corresponding standards. Quantification was carried out using calibration curves for each compound analyzed using concentrations between 250–2.5 mg/mL (250, 125, 100, 50, 25, 10, 5, 2.5 mg/mL). In all cases, the coefficient of linear correlation was *R*^2^ > 0.99. Compounds were quantified and identified at different wavelengths (209–370 nm).

#### 2.4.4. ATR-Fourier Transform Infrared Spectroscopy

Chemical groups and bonding arrangement of constituents present in the PBE dried samples were determined by Fourier Transform Infrared Spectroscopy (FTIR) using an ALPHA II- Bruker spectrometer (Ettlingen, Germany) with a diamond-composite attenuated total reflectance (ATR) cell. The measurements were recorded with a wavenumber range from 4000 to 400 cm^−1^, with a resolution of 4 cm^−1^ and 24 scans per sample.

### 2.5. Evaluation of in Vitro Bioactivities

#### 2.5.1. Antioxidant Activity

Three different methods of measuring the antioxidant activity were used: DPPH, ABTS and FRAP, as previously described by Ferreira-Santos et al. [5].

Free radical scavenging assay (DPPH assay) consists in the reduction of the 2,2-diphenyl-1-picryl-hydrazyl-hydrate (DPPH^•^) radical in the presence of hydrogen-donating antioxidant, and in the formation of the non-radical DPPH-H form at the end of the reaction.

Scavenging activity of 2,2′-azino-bis-(3-ethylbenzothiazoline-6-sulfonic acid) radical cation (ABTS^•+^) (ABTS assay) is based on interaction between antioxidant and ABTS radical, that, in the presence antioxidant compounds, the ABTS^•+^ nitrogen atom quenches the hydrogen atom, causing the solution decolorization.

The concentration of the tested PBE and Trolox as a standard compound ranged between 1 and 250 μg/mL. The lyophilized extracts were re-suspended in the respective solvent used in the extraction process, and the Trolox prepared in absolute ethanol. A corresponding control was used for each solvent.

The radical scavenging activity for DPPH and ABTS methods (% inhibition) was calculated as Equation (2).

(2) $$\%\;\mathrm{Inhibition}=\frac{\mathrm{Ac}-\mathrm{As}}{\mathrm{Ac}}\times 100$$

where As represents the sample absorbance and Ac the control sample absorbance. The results were expressed as the sample concentration (µg/mL) required to inhibit 50% of the activity (IC_50_) calculated from a dose response curve using GraphPad software (San Diego, CA, USA).

Ferric reducing antioxidant power (FRAP assay) consists in the ability of extracts to reduce ferric ions (Fe^3+^ to Fe^2+^), in the form of ferric 2,4,6-tripyridyl-s-triazine (TPTZ). FRAP values are expressed as micromoles of ferrous equivalent per g of dry weight (µmol Fe^2+^/g PBE).

#### 2.5.2. Antihyperglycemic Activity

Two different methods of measuring the potential antihyperglycemic activity were used: α-Amylase and α-Glucosidase inhibition assays. These methodologies were previously reported by Irondi and coworkers [22], and used with some minors modifications. The concentration of the tested PB extracts (dissolved in 20% DMSO and 80% water) were between 50–1000 μg/mL for α-Amylase assay (1000, 750, 500, 250, 125, 50 μg/mL) and 1–250 μg/mL for α-Glucosidase assay (250, 200, 150, 100, 50, 25, 10, 1 μg/mL, prepared by successive dilutions).

For the α-Amylase inhibition assay, different concentrations of PBE were incubated with α-amylase (0.5 mg/mL) and 1% of starch solution for 15 min at 37 °C. Afterwards, dinitrosalicylic acid color reagent (96 mM 3,5-dinitrosalicylic acid, 5.31 M sodium potassium tartrate in 2 M NaOH) was added to the reaction and placed 10 min in a boiling water bath. This heating step also allowed to stop the reaction (inactivate the enzyme). Finally, the mixture was diluted 10 times in distilled water. In these conditions, the reduction of 3,5-dinitrosalicylic acid to 3-amino-5-nitrosalicylic acid allows to quantify maltose which being detectable at 540 nm. Acarbose was used as a reference control, and the concentration used for a-amylase assay is between 1–200 µg/mL (200, 100, 50, 25, 10, 1 µg/mL). The a-amylase inhibition (%) was calculated using the same equation (Equation (2)) used for DPPH assay.

For α-Glucosidase inhibition assay, α-Glucosidase solution (10 U/mL) was incubated with different concentrations of PBE and p-nitrophenyl-R-d-glucopyranoside (pNPG, 3 mM). The mixture was then incubated at 37 °C for 15 min, and the reaction was stopped by adding Na_2_CO_3_ solution (1M). The activity of α-glucosidase was determined by measuring the absorbance of *p*-nitrophenol released at 400 nm. Acarbose was used as a reference control, and the concentration used for α-glucosidase assay is between 2500–15,000 µg/mL (15,000, 12,500, 10,000, 7500, 5000, 2500 µg/mL). The α-glucosidase inhibitory activity was calculated using the same equation (Equation (2)) used for DPPH assay.

The results were expressed as the sample concentration (µg/mL) required to inhibit 50% of the activity (IC_50_) calculated from a dose response curve using GraphPad software.

#### 2.5.3. Antimicrobial Activity

For this assay, a disk diffusion method was used to determine the diameter of the inhibition zone of tested extracts (50 mg/mL of each PBE´s reconstituted in DMSO (≥99.9%)) and was performed following the protocols established by the Clinical and Laboratory Standards Institute (2012) [23]. The PBE concentration was selected based on the results obtained from preliminary experiments (data not shown) and data from the literature in which extracts rich in polyphenols were used [24].

Strains of Bacillus cereus ATCC 11778, Clostridium perfringens ATCC 13124, Escherichia coli ATCC 25922, Listeria monocytogenes ATCC 13932, Staphylococcus aureus ATCC 25923, Salmonella enterica serovar Enteritidis ATCC 25928, Aspergillus brasiliensis ATCC 16404, Saccharomyces cerevisiae NCTC 10716 and Candida albicans ATCC 10231 were inoculated in Columbia Agar + 5% Sheep Blood (COS, Biomérieux, Craponne, France). Active cultures (0.5 McFarland) were spread onto Mueller-Hinton Agar (MHA) (Oxoid, Basingstoke, England) for general bacteria and MHA + 0.2% glucose for fungus. Blank disks 6 mm in diameter were placed onto inoculated plates and impregnated with 10 µL of PBE or controls (DMSO and commercial solution of sodium hypochlorite (LX)). In this study, a solution of sodium hypochlorite was used as positive control instead of commercial antibiotics, in order to control the sensitivity of the microbial test and reduce antibiotic use in basic research studies. Afterwards, the plates were incubated for 24 h at 37 °C (for bacteria) and 48 h (for yeasts and fungi). Zones of inhibition were measured in mm with the help of ImageJ software (US National Instiutes of Health, http://rsb.info.nih.gov/ij/).

#### 2.5.4. Cell Viability

In vitro cell metabolic activity of the PB extracts was assessed in different cell lines: normal mouse fibroblast (L929-ATCC^®^ CCL -1), human embryonic kidney (HEK293T- ATCC^®^ CRL-11268) and human lung cancer (A549- ATCC^®^ CCL-185); these cell lines were kindly provided by Andreia Gomes (Department of Biology, University of Minho). The metabolic activity of each cell line was evaluated by the resazurin reduction assay [25]. Cells were grown Dulbecco’s Modified Eagle Medium (DMEM) supplemented with 10% fetal bovine serum (FBS) and 1% penicillin/streptomycin, at 37 °C in a humidified atmosphere with 5% CO_2_. When the cell culture reached 70–80% of confluence, the cells were trypsinized and seeded in a 96-well plate at a density of 1 × 10^5^ cells per well. The different cell lines were incubated with supplemented DMEM and PBE in a concentration ranging from 75 to 1000 µg/mL for 24 h. After incubation, cell viability was measured using the resazurin assay (7-Hydroxy-3H-phenoxazin-3-one-10-oxide sodium salt). The supernatant was replaced by 200 μL culture media containing resazurin (0.5 mM in PBS). After 2 h of incubation at 37 °C, 150 μL of the supernatant were transferred to a new 96-well microplate and the pink fluorescent resultant product (resorufin) was detected at 560 nm (λex) and 590 nm (λem) using a microplate reader (Cytation 3, BioTek Instruments, Inc., Winooski, VT, USA).

The % cell viability was calculated correcting blank values (cell-free medium) and related to untreated controls (0.5% DMSO). IC_50_ values were calculated from a dose response curve using GraphPad software.

### 2.6. Statistical Analysis

All experiments were performed in triplicate and the data are presented as mean ± standard deviation (SD) values. GraphPad Prism^®^ software (version 6.0; San Diego, CA, USA) was used for statistical analyses. The analysis of variance (ANOVA) and Tukey’s multiple comparisons test were used to determine statistically different values at a significance level of *p* < 0.05.

## 3. Results and Discussion

### 3.1. Chemical Characterization of PB

The chemical composition of the PB is summarized in Table 2. The results show that the majority fractions of the total bark composition are lignin, representing 41.6% (being 41.0% klason and 0.6% acid soluble lignin). The monomeric composition of polysaccharides, which correspond to an average of approximately 30% of pine bark, shows a predominance of the cellulose fraction (17.4% of glucose content). In the hemicellulose fraction, xylans are most representative (10.9%), being arabinan and mannan groups present in 1.4% of total monosaccharides.

In this work, the ethanol soluble extractives represent 13.2% of total PB composition.

The inorganic substances represent 2.6% of total bark composition, and the most relevant minerals, determined by plasma atomic emission spectrometry, are potassium, magnesium, calcium and iron.

Other constituents such a lipid fraction (fat) and protein content represent 2.5% and 1.6% of total composition of PB, respectively.

### 3.2. Solid–Liquid Extraction

#### Influence of Variables on the TPC and Antioxidant Activity

Assays were performed using water and ethanol (30%, 50%, 70% and 90% (*v*/*v*)) as solvents, in order to select the best extraction conditions for each solvent. In this way, five different extracts were generated with different polarities and features presumably representative of the pine bark potential. These extracts were used in the subsequent analyzes.

The solvents used (water and ethanol) are considered natural, environmentally friendly (green solvents), nontoxic and food grade [16,26]. This is very important, as the extracts obtained will be studied as possible food and nutraceutical applications for human or animal consumption.

The influence of independent variables, such as time, temperature and solid-liquid ratio for different ethanol concentration on the TPC and antioxidant activity (FRAP), was evaluated by the models shown in Table 1 and in Supplementary Materials Figure S1 (Contour line plots for FRAP variable) and S2 (Pareto chart). The significance of each coefficient was also determined using *F-value* and the corresponding *p*-value (Supplementary Materials Table S1).

Statistical analysis for each solvent (water (EtOH 0%) or water-ethanol mixtures) is represented in Supplementary Materials, Figure S2 and Table S1.

The experimental variables were correlated following Equation (1) (quadratic model). The proposed mathematical models describing the extraction time (x_1_), temperature (x_2_) and solid-liquid ratio (x_3_) as function, and using normalized values of regression coefficient for TPC and FRAP, are presented in the Supplementary Materials Table S2. In addition, the validation experiments performed under the predicted conditions derived from the experimental design demonstrated that the experimental values were close to the predicted values (data not shown), confirming the validity and adequacy of the proposed mathematical models.

The conditions of test number 8 of all experimental models, which extraction conditions of 115 min, 82 °C and a solid-liquid ratio of 0.15 g/mL (6 g /40 mL), showed a higher TPC (48.1 mg GAE/g PB for EtOH 0%, 120.1 mg GAE/g PB for EtOH 30%, 163.6 mg GAE/g PB for EtOH 50%, 136.5 mg GAE/g PB for EtOH 70%, 123.8 mg GAE/g PB for EtOH 90%, respectively) compared to the other conditions tested. In addition, the antioxidant activity evaluated by the FRAP method was also in agreement with the TPC of the obtained extracts. Taking into account these results to obtain extracts with high content of bioactive polyphenols, the conditions mentioned above were selected in order to evaluate the chemical profile, potential bioactivity and cytotoxicity of the extracts.

### 3.3. Phenolic and Flavonoid Contents of PBE

**Figure 1:**
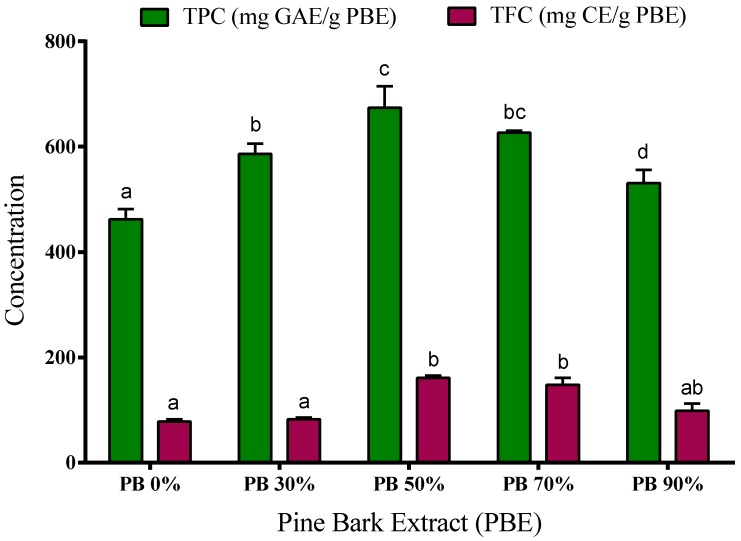
Total phenolic content (TPC) and total flavonoid content (TFC) of aqueous and hydroethanolic extracts from *Pinus pinaster* bark. Values are expressed as mean ± SD of 3–4 experiments; GAE: gallic acid equivalents; CE: catechin equivalents. Different letters show significant differences (*p* < 0.05) between groups for the same experiment.

The spectrophotometric determination of total phenolic content (TPC) and total flavonoid content (TFC) of aqueous and hydroethanolic extracts from *P. pinaster* bark are presented in Figure 1. Moreover, the individual phenolic compounds were identified and quantified using liquid chromatography, and the results are presented in Table 3.

The use of specific solvents is responsible for the selectivity of the compounds in the extract, and the dissolution of intracellular compounds of the raw material, i.e., plants or by-products [27]. Important metabolites with antioxidant properties of natural resources, such as phenolic compounds, are more soluble in polar solvents, due to the presence of a hydroxyl group [27,28]. Therefore, in this work, water and ethanol were used as effective environmentally friendly polar solvents [16].

Our results show that the PB extracts have a high content of phenolic compounds. The phenolic content was determined by the Folin–Ciocalteu method, ranging from 460 to 675 mg of GAE/g PBE. The extracts obtained using 50% and 70% ethanol have higher concentration of TPC (674.5 ± 23 and 626.8 ± 2 mg GAE/g, respectively) when compared to aqueous, 30% and 90% hydroalcoholic extracts (462.5 ± 11, 586.5 ± 11 and 530.8 ± 15 mg GAE/g, respectively). Even so, water extracts present a very significant number of phenolic compounds showing high bioactive or functional potential, and water should be considered as a promising solvent due to its greener character.

The extracts’ flavonoid content ranged from 77 to 161 mg CE/g PBE depending on the solvent used in the extraction process. The highest content was observed in the 50% and 70% ethanol extracts (161 ± 3 mg CE/g and 148 ± 9 mg CE/g, respectively), while the extracts obtained with water, 30% and 90% of ethanol showed the lowest TFC (77.5 ± 4 mg CE/g, 81.9 ± 3 mg CE/g and 98.6 ± 9 mg CE/g, respectively) (*p* < 0.05).

These results are in accordance with those described previously by our research group, where it was observed that *P. pinaster* bark extracts are very rich in phenolic compounds [5]. Moreover, Chupin et al. [12] use *P. pinaster* bark and reported values from 236 to 306 mg GAE/g extract, depending on the extraction conditions, but the maximum was achieved with 80% ethanol. In another study [29], the values of TPC are between 22–62 mg GAE/g bark, results that are in agreement with those obtained in our study (30.75, 54.0, 68.2, 65.1, 57.9 mg GAE/g bark for H_2_O, EtOH 30%, EtOH 50%, EtOH 70%, EtOH 90%, respectively). Also, extracts from other species of pine [4,30,31] (*Pinus roxburghii*, *P. wallichiana*, *P. radiata*, *P. gerardiana*, *P. mariana* and *P. banksania*) showed high content in TPC and TFC. For example, Sharma et al. [4] obtained hydroethanolic extracts (90% ethanol) from bark of three different pine species (*Pinus roxburghii*, *P. wallichiana* and *P. gerardiana*), and the extracts showed TPC concentrations between 222 to 249 mg GAE/g extract and high TFC content (477–597 mg rutin equivalents/g extract). The study by Royer et al. [30] demonstrated that aqueous and hydroethanolic extracts (95% ethanol) of Canadian pine species have TPC values between 27 to 346 mg GAE/g extract and 6–39 mg quercetin equivalents/g extract for TFC, values below those obtained in our work for *P. pinaster* bark.

The ratios of 50:50 and 30:70 (*v*/*v*) water-ethanol were the ones presenting the highest TPC and TFC content. If the ethanol concentration used on the extractive process is higher (90% *v*/*v*), the extracts contain lower amounts of these secondary metabolites. Other research works also reported higher phenolic compounds extraction when intermediate ethanol concentrations are used as solvents for other extraction matrices [32,33]. Jiménez-Moreno and co-workers [32] studied different extraction conditions to obtain grape stem extracts rich in polyphenols, and their results showed that using 50% ethanol the extraction is maximized and the extracts show greater antioxidant activity. Recently, Tanase et al. [34] gathered bibliographic information on the extraction of phenolic compounds from the bark of woody plants and their potential biological activity, showing that several solvents are used to obtain phenolic compounds. In this review, the authors report that the mixture of water with ethanol has been widely used to maximize the extraction of these compounds derived from lignocellulosic matrices, such as the *Pinus* species. This may have to do with the ability of the solvent to penetrate the cell wall, allowing the phenolic compounds to escape [32].

Chromatographic analysis of extracts was performed to identify and quantify the individual compounds and to evaluate the selectivity of each solvent used in the extraction process over the chemical profile (phenolic composition) of the extract (see Table 3).

A total of 18 polyphenol compounds belonging to the groups of acids, flavan-3-ol, flavonoids and stilbens were identified according to their corresponding standards. The extraction with hydroethanolic mixtures resulted in the highest extraction of almost all phenolic compounds, except for the gallocatechin, which was only detected in aqueous and 30% ethanol extracts (149.3 and 140.3 mg/L, respectively).

All extracts contain high concentrations of taxifolin and ellagic acid, which increase as the ethanol concentration increases in the extraction process. Taxifolin was found in concentrations ranging from 73.1 mg/L to 463.9 mg/L (representing 21–42% of the total phenolic composition) and ellagic acid concentration was 53.6–124.4 mg/L (9–16% of the total phenolic composition). Narigenin and catechin have only been identified and quantified in hydroethanolic extracts, representing an average of 20% and 13% of the extract composition, respectively. Taxifolin was also one of the main constituents of pine seed extracts from *P. sibirica* [35]. Catechin and taxifolin were found in extracts of pine barks from five different species *P. pinea*, *P. sylvestris*, *P. nigra*, *P. parviflora*, and *P. ponderosa* harvested from different locations [36]. The authors concluded that higher concentrations of catechin and taxifolin are due to climatic stress conditions like less rainfall, higher temperatures and longer duration of sun exposure. However, taxifolin was not found in *P. nigra*, and *P. sylvestris* needle extracts harvested in Macedonian flora [37]. Quercetin has been identified in extracts obtained with the highest concentration of ethanol (i.e., 50% to 90% (*v*/*v*)) having concentration values of 8.5 to 10.9 mg/L. Ferulic acid was found in extracts of spruce bark in higher concentrations (between 23.9 and 28.6 mg/g) than presented here [38]. Epicatechin, *p*-coumaric acid, gallic acid and hesperidin were identified in all samples, but could not be quantified because they coeluted. Epicatechin, *p*-coumaric acid were found to be the main constituents in the extracts of pine needles [39].

Though the phenolic composition of pine extracts may depend on variables like pine species, used solvent, environmentally conditions and geographical location, the detected and determined individual phenolics of PBE are in agreement with data from our previous studies [5] and the literature results [12].

Polyphenols are very diverse compounds, with different polarities and chemical characteristics. Their extraction depends on several factors, such as solvent to matrix ratio, type of matrix, extraction method, extraction time, extraction temperature, extraction solvent, among others. The solvents with different polarities proportioned extracts with different amounts or types of constituents [40]. More polar solvents are expected to extract more polar molecules [40]. For example, the extraction of hydrophilic compounds uses polar solvents such as methanol or ethanol, and for extracts, more lipophilic compounds, dichoromethane or mixtures with methanol are used. This affinity for the solvent will determine also the solubility of the different compounds, and may limit the maximum possible extractable amount [41]. In this context, the results obtained are within the expected. Flavonoids have very poor solubility in water. Even though they may have oxygen atoms for hydrogen bonding with water in side chains, they have a relatively large and complex structure with a high number of carbon atoms which gives them the strong apolar character [42]. Very poorly soluble in water compounds, such as cathechin, naringenin, taxifolin, quercetin or resveratrol, are almost not detected when water is used as solvent. However, their solubility can be strongly increased in the presence of ethanol, which reflected in the much higher amounts extracted particularly above 50% in ethanol content. However, this increase was not visible for higher amounts of ethanol, in particular when comparing the results from 70% and 90% ethanol. This may indicate that there is a balance to be made between the affinity of the solute towards the solvent and the ability of the solvent to enter in the matrix structure. As referred to above, this last one seems to decrease for very high amounts of ethanol. On the other hand, smaller phenolics such as 3,4-dihydroxybenzoic acid, caffeic acid or ferulic acid have higher (though still poor) solubility in water, with the differences between using just water or hydroethanolic mixtures as a solvent being not so sharp.

This detailed analysis of the chemical composition of PBE obtained with non-toxic solvents allows us to state that these natural extracts are rich in phenolic acids, flavan-3-ols, flavonoids and stilbene compounds, that could be used as high added value products, i.e., a viable alternative for food and nutraceuticals industry applications.

### 3.4. ATR-FTIR Spectra Analysis

**Figure 2:**
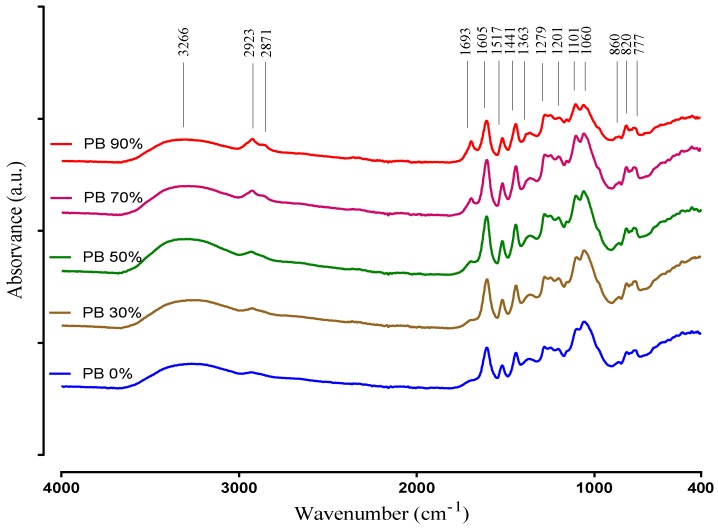
ATR-FTIR spectra of aqueous and hydroethanolic extracts from *Pinus pinaster* bark.

The ATR-FTIR technique was used to obtain information regarding the major functional groups present in the plant extract samples. The spectra of the PB compounds extracted with different solvents are recorded in the 400–4000 cm^−1^ region and are presented in Figure 2.

The IR spectra interpretation was performed according to Chupin et al. [12], Coates [43] and Ricci et al. [44], and clearly shows differences between the PBE´s corroborating the results obtained in the other chemical determinations (i.e., TPC, TFC and UPLC-DAD). The very broad band observed at 3600–3000 cm^−1^ is assigned to the hydroxyl compounds, O–H stretching vibration in phenolic and aliphatic structures. Other characteristic vibration that is observed in this region is the C–H stretch of a terminal alkyne. It exhibits a relatively narrow absorption at 3300 cm^−1^. Small peaks at 2923 and 2871 cm^−1^ originate from C–H stretch vibration in methyl groups. These peaks are clearly more intense in the extracts when obtained with the highest ethanol concentrations, and could be attributed to hydrophobic phenolic compounds extracted with this conditions. Nevertheless, these peaks may also be related with a higher amount of lipids that may be co-extracted in these conditions, as they seem to increase up to the 90% ethanol solvent (while most phenolics decrease from 70% to 90%).

Absorption in the region 1850–1650 cm^−1^ usually indicates the presence of a C=O group (carbonyl compound). On the other hand, the band identified at 1693 cm^−1^ is attributed to carbonyl stretch and it is characteristic of hydrolysable tannins. It is obvious that this peak is increasing with the increase of ethanol. Compounds that have C=O are better extracted with ethanol. The peaks at 1605 cm^−1^, 1517 cm^−1^ correspond to aromatic skeleton vibrations and to –CH deformation at 1440 cm^−1^. Additionally, the peak at 1517 cm^−1^ indicates the presence of non-gallate procyanidins. These structures identified by the presence of the previous peaks indicate the presence of phenolic compounds in the PBE samples. Therefore, in the spectra relative to the PB extracts 30%, 50% and 70% are presented with a higher intensity than in the spectra of the samples PB 0% and PB 90%, according to the results of TPC and TFC. The band at 1363 cm^−1^ is attributed to phenolic stretch vibration of –OH and aliphatic –CH deformation in methyl groups. The bands located at 1201 cm^−1^ and 1060 cm^−1^ represents the asymmetric and symmetric stretching vibrations of –CO and aromatic –CH bending in plane bending vibrations detected at 1101 cm^−1^ demonstrating aromatic ring deformations and interactions with rings substituents, due to phenols and flavonoids structures. The peaks intensity differences of PBEs at 1101 cm^−1^ and 1060 cm^−1^ can be due to an opening of the cyclic ether structure of phenols. Peaks between 860 to 777 cm^−1^ show the stretching and bending vibrations of –CH from aromatic rings, related to phenolic compounds. This is also supported by the peaks in the range 1600–1500 cm^−1^, and the locations of the bands are often indicative of the nature of the substitution of the aromatic ring. In simple structures, it is possible to differentiate mono- and di- (ortho, meta and para) substitution. For example, the peak 860 cm^−1^ indicates for para substitution. That is in accordance with the identification of the individual phenolic compounds, as all samples had *p*-coumaric acid.

### 3.5. Antioxidant Activity of PBE

The antioxidant capacity of natural molecules and extracts is one of the most studied biological activities, being referenced as a mechanism to prevent the oxidative stress and several diseases. Moreover, the antioxidant activity of the plant extracts depends on the composition and structure of the bio-compounds, such as a phenolic acids and flavonoids and their ability to neutralize Reactive Oxygen Species (ROS) and other free radicals, i.e., chelators and free radical scavengers activities [45].

In the food context, the emergence of new antioxidant sources has been the target of recent research and applications in food protection and nutritional enrichment, promoting the consumption of bioactive compounds.

In this sense, different methods were used for the determination of antioxidant activity, allowing the analysis of different mechanisms of extracts action. The ferric reducing antioxidant power (FRAP) method is based on the reduction of an iron complex (Fe^3+^ to Fe^2+^), and DPPH and ABTS are the most widely used method for determining the free radical scavenging capacity [7,46].

The results of DPPH, ABTS and FRAP for the PBE are represented in Table 4. Our data shows that the extracts obtained with 50% ethanol (PB 50%) have the highest radical scavenging activity (IC_50_ value of 49.74 µg/mL for DPPH and 59.41 ± 2.1 µg/mL for ABTS) (*p* < 0.05). The other extracts also have a high inhibition percentage, obtaining an increase on the IC_50_ values for PB 70%, PB 30%, PB 0% and PB 90%, respectively. This is in accordance with previous statement that ethanol have an important role in the extraction of compounds with higher antioxidant activities [13].

The FRAP assay also proved the potent reducing power of pine bark extracts, and the results are in agreement with those obtained in the DPPH and ABTS methods. The PB 50% extract shows the greatest reducing power (138.5 mmol Fe^2+^/g PBE), followed by extracts PB 70% (122.9 mmol Fe^2+^/g PBE), PB 30% (112.4 mmol Fe^2+^/g PBE), PB 0% (101.9 mmol Fe^2+^/g PBE) and lastly the extract PB 90% (101.3 mmol Fe^2+^/g PBE).

These results show the correlation of the total phenolic and flavonoids content of the bark extracts with the antioxidant activity measured by the DPPH, ABTS and FRAP assays, demonstrating the contribution of these extracted bio-compounds to the antioxidant activity.

Recently, Gascón and coworkers [47] proved the high antioxidant activity of phenolic extracts from *P. pinaster* bark using in vitro methods, i.e., ABTS (2,2′azino-bis-(3-ethylbenzoithiazolone-6-sulphonic acid)) and in the control of cellular ROS in Caco-2 cells. Also, our group reported that extracts obtained from this by-product using different extraction methods have a large antioxidant capacity, determined by the DPPH, FRAP and ABTS methods [5]. Moreover, the total phenolic compounds of the pine bark extracts were highly correlated with their antioxidant power [5]. Other authors also reported the potential antioxidant activity of *P. pinaster* extracts. Yesil and co-authors [36] also found a high correlation between the antioxidant activity of PBE and its total phenolic content, as well as its total amount of constituents. Moreover, the author demonstrated that the DPPH values of PBE of the same species harvested from different locations did not show considerable differences in comparison to the total phenol values. In another study, the authors managed to connect the increment in the antioxidant activity of extracts with the increment in catechin + epicatechin contents, which were the only phenolic compounds measured [13].

### 3.6. Antihyperglycemic Activity

In recent years, compounds of natural origin, such as phenolic compounds, have been extensively studied for their high biological activity. Among the most prominent activities of phenolic compounds are the activity of free radicals’ inhibition, antibacterial, anticancer, etc., and more recently the activity of inhibiting digestive enzymes, such as α-amylase and α-glycosidase, involved in the pathological state of hyperglycemia associated with diabetes mellitus. Inhibition of these enzymes blocks carbohydrate hydrolysis and decreases glucose absorption in the body [4,22,48,49].

In this sense, PBEs were studied to find out if they had inhibitory activity of these enzymes, and the results are presented in Table 4. The obtained results showed that all of the extracts, regardless of the solvent used in the extraction, have α-amylase (IC_50_ values between 254.2 ± 9.2 to 576.4 ± 2.9 µg/mL) and α-glycosidase (IC_50_ values from 122.74 ± 11.3 to 166.22 ± 1.1 µg/mL) inhibitory activity. It is possible to observe that the extracts obtained with highest concentrations of ethanol (70% and 90% *v*/*v*) have a higher α-amylase inhibition (*p* < 0.05), presenting lower IC_50_ values. On the other hand, the hydroethanolic extracts obtained with 30%, 50% and 70% ethanol (PB 30%, PB 50% and PB 70%, respectively) showed the greatest inhibition in α-glucosidase activity.

These results indicate that PB extracts have a potent inhibition of digestive enzymes, and may be considered when seeking for alternatives in the prevention or treatment of diabetes.

Both methods used to evaluate the antihyperglycemic activity of the extracts were validated using a commercial inhibitor, acarbose. In the α-amylase assay, acarbose has an IC_50_ of 35.42 ± 1.0 µg/mL, noting that all extracts have less activity than the commercial inhibitor. When activity is evaluated using α-glucosidase, acarbose has IC_50_ values of 11.00 ± 1.0 mg/mL, much higher than those obtained in PB extracts. The authors Irondi [22], Bezerra [50] and their collaborators, also reported a decrease in the α-amylase and α-glucosidase inhibitory effects using doses of acarbose similar to those presented here.

Schäfer and Högger [51], and Liu and coworkers [52] conducted clinical studies on diabetes patients using a commercial aqueous pine bark extract (Picnogenol^®^, Horphag Research, Geneva, Switzerland), and reported a potent antidiabetic activity of the procyanidin-rich extract. In another work, authors studied the effect of PBE (70% EtOH) on diabetic mice and observed an inhibition effect against salivary α-amylase and yeast α-glucosidase, decreasing carbohydrate absorption [53].

These results show that aqueous and hydroethanolic extracts from *P. pinaster* bark have the potential to reduce glucose absorption, and can be used as a food supplement with antidiabetic properties.

### 3.7. Antimicrobial Activity

It is known that the aqueous and ethanolic plant extracts may exhibit antimicrobial activity. In this sense, the screening of antibacterial and antifungal activities of PBE´s were assessed by the agar well diffusion plate method, by estimating the diameter of zone of inhibition against Gram-positive (*S. aureus, C. perfringens*, *L. monocytogenes and B. cereus*) and Gram-negative (*E. coli*, *Salmonella* Enteritidis) bacteria, two yeasts (*C. albicans* and *S. cerevisiae*) and a fungi, *A. brasiliensis*. These microorganisms were selected due to the well-known causes of these pathogens to foodborne diseases, except *C. albicans* [54]. The diameter of zone of inhibition by all extracts is presented in Table 4.

Generally, Gram-negative bacteria are more resistant than Gram-positive bacteria. Therefore, it was important to evaluate the antimicrobial properties of PBEs obtained under different conditions and the in presence of different of bacteria, yeast and fungi strains.

The results of PBEs demonstrate a potent antibacterial activity against Gram-positive bacteria. At the concentration of 50 mg/mL, the highest inhibition zones were achieved against *C. perfringens*, and the most resistant bacteria was *L. monocytogenes*. These extracts did not show activity for Gram-negative bacteria, nor antifungal activity against *C. albicans*, *S. cerevisiae* and *A. brasiliensis*.

It is apparent that the extracts obtained by hydroethanolic extraction have a higher antibacterial activity than the aqueous extracts. This may be due to the higher contents of phenol and flavonoids in these extracts (Figure 1 and Table 3).

While plants serve as rich, natural and safer sources of antimicrobials, the rapid incidences of increased resistance to available antibiotics worldwide have turned the attention of researchers and the pharmaceutical industries to plants in search of viable alternatives.

There are different studies that demonstrates the antimicrobial activity of the phenolic compounds [55], more specifically, plant bark extracts used as a natural preservatives. In a previous study, different polyphenolic bark extracts from Canadian forest species and a commercial product from pine bark were tested against two non-pathogenic bacteria strains (*E. coli* and *L. ivanovii*). Their results show that these extracts were more active for Gram-positive bacteria [30]. These results are consistent with the data obtained in our study.

Natural extracts, such as PBEs, rich in antioxidant bioactive compounds like phenolics (phenolic acids and flavonoids) can be a viable alternative to the serious problem of microbial resistance to antibiotics. In this sense, PBEs are a good option to be considered as a complement to bio-preservation for the food industry, particularly in foods where Gram-positive bacteria are important and common contaminants.

### 3.8. Cell Viability

As demonstrated, the PBE has enormous potential to be used for human consumption and as a nutraceutical component. Therefore, the next step is to verify in which concentrations the extract is safe to be used. For that three different cell lines, one normal mouse cell line (L929) and two human cell lines (normal—HEK293T—and derived from cancer tissue—A549) were used. Several authors have used similar cell lines to evaluate the potential toxicity of other plant extracts and isolated products [56,57]. Different concentrations of active extracts were placed in contact with the cells and their ability to metabolize resazurin into resorufin in the presence of PBE was used as a measurement of cells metabolic activity. A decreased of resazurin conversion indicates impairment of cellular metabolism [25], being an indication of the PBE toxicity.

The cells were exposed to different concentrations of each extract for 24 h (0 to 1000 µg/mL). The three cells lines demonstrated a dose-dependent effect. However, the behavior of non-tumor cells was different from the behavior of tumor cells. The presence of low concentrations of all extracts (75 and 125 µg/mL) stimulated their metabolic activity, while, for the same concentration, the tumor cells had a decrease on their metabolic activity. Though the variability of results is high (which is common in these type of tests), this effect is statistically different and this result is in accordance with the literature, where it is described that naturally available extracts of different sources selectively inhibit abnormal cell proliferation without interrupting normally functioning cells [58]. Therefore, this finding is the ideal condition for a potential anti-cancer effect, as it would only affect the cancer cells and not the healthy cells. However, this is still a very preliminary indicator (useful in a first potential screening) and further tests at different levels (in vitro, ex vivo and in vivo) are needed to validate (or not).

**Figure 3:**
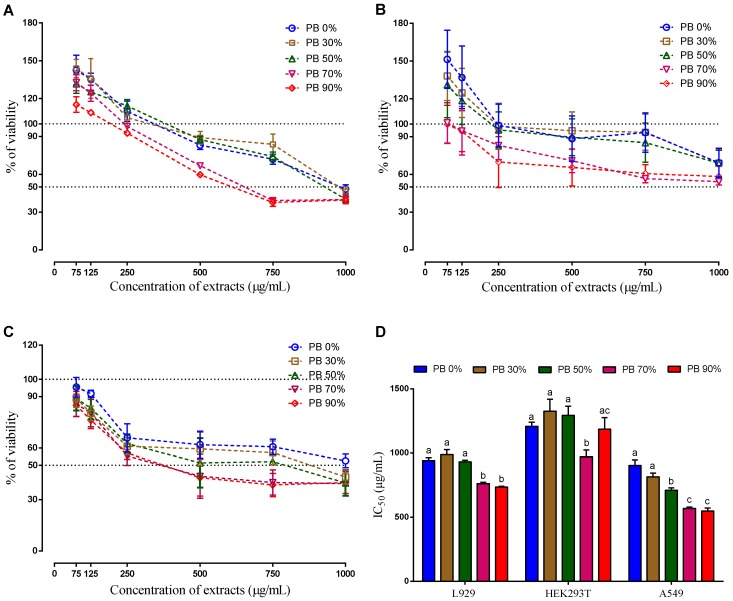
Cellular viability (%) of aqueous and hydroethanolic extracts from *Pinus pinaster* bark against normal mouse fibroblast (L929) (**A**), human embryonic kidney (HEK293T) (**B**), human lung cancer (A549) (**C**) cell lines, and the respective IC_50_ values (**D**). Values are expressed as mean ± SD of 4-5 experiments. Different letters show significant differences (*p* < 0.05) between groups for the same experiment.

When analyzing the IC_50_, it is clear that the action mechanism of the compounds present in the PBE extracts is different for the three cells lines. As it can be seen on Figure 3 the IC_50_ decreases with the increase of ethanol on the extraction solvent for the mouse cells line (L929) and for the human cancer cell line (A549), on the hand, there is no clear relationship between the IC_50_ and the solvent extraction composition for the human normal cells line (HEK 293T). As it is known the solvent extraction composition influences the extracted compounds. The highest IC_50_ for the non-tumor cells was observed for the PBE extracted using 30% of ethanol (PB 30%), while for the tumor cell the highest is for aqueous (PB 0%), followed by PB 30%. Interestingly the extract PB 30% does not have the highest content in TPC or TFC, which indicates that the total amount of these compounds is not the main reason the cellular mechanisms observed. Even when comparing with the DPPH, ABTS and FRAP, there is no clear evidence. The extracts PB 0% and PB 90% present similar anti-oxidative activity. Moreover, these extracts showed the lowest anti-oxidative activity comparatively to the rest of the extracts. Therefore, it is not clear which mechanism was responsible for the observed cell behavior. Moreover, the antioxidant effect of PB extracts on the oxidative status of these cancer cell lines should be studied in the future.

When analyzing the composition of the extracts, it is possible to observe that gallocatecchin is only present in PB 0% and PB 30% and quercetin is absent (or undetected), only being detected on the PB 50%, 70% and 90% that induced the highest cytotoxicity (lower IC_50_), except for the non-tumoral cell line HEK293T.

It has been described that gallocathecin can inhibit the tumor cell line HCT-116 growth up to 57%, and that quercetin can also induce selective growth inhibition and apoptosis in hepatic tumor cells, but not in normal cells [59]. Also, the quercetin showed the strongest dose-dependent anti-proliferative activities to colon cancer cells (HT-29) and liver cancer cells (HepG2) [46].

Taking in consideration the results obtained, it is hypothesised that the selectivity of the PBE obtained are related to a synergetic effect of several molecules, in particular to gallocathecin and quercetin.

Recently, Gascón and coworkers [47] described the antiproliferative, apoptotic and redox system controlling effects of bark extracts from three pine species, including *Pinus pinaster*, on Caco-2 cells. Touriño and collaborators [60] reported that *P. pinaster* bark extracts have a high antioxidant activity and can control proliferation in a human melanoma cell line. Additionally, Mao et al. [57] demonstrated that extracts from the *Pinus massoniana* bark inhibit migration of the lung cancer A549 cell line.

These results demonstrate that the aqueous and hydroethanolic extracts of *P. pinaster* bark at the tested concentrations present low cytotoxicity, and may have the potential to inhibit the tumor cell growth to some extent, though this has to be further validated.

## 4. Conclusions

The present study revealed a wide range of phytochemicals in *P. pinaster* extracts, belonging to different chemical groups: phenolic acids, flavonoids, flavonols and stilbens. Extracts showed different phenolic profiles, depending on the solvent used. However, all demonstrated high potential antioxidant, antidiabetic and antimicrobial activities. The extracts from intermediate ethanol concentrations (50% and 70%) showed the highest bioactivities. These features demonstrate the potential of these extracts to be used in food formulation and processing, either with a technologic function (such as preservative or antioxidant) or as a bioactive ingredient. Moreover, the PBEs have low cytotoxicity, but most importantly, they act selectively on cancer cells, as these are negatively affected and the non-tumor cells are not. These results unravel the potential use of PBE in the medical or nutraceutical sectors.

## Figures and Tables

**Table 1:** Experimental runs using coded levels of time (min, x_1_), temperature (°C, x_2_) and solid: liquid ratio (g/mL, x_3_) according to the 2^3^ full factorial central composite design and data of total phenolic content (TPC) and reducing antioxidant activity (FRAP) of extracts obtained under those conditions for the tested experimental model (EtOH 0%, EtOH 30%, EtOH 50%, EtOH 70% and EtOH 90%).

Runs	Time (min) *x_1_*	Temperature (°C) *x_2_*	Solid-Liquid Ratio (g/mL) *x_3_*	EtOH (*v*/*v*)									
				0% (H_2_O)		30%		50%		70%		90%	
				*TPC*	*FRAP*	*TPC*	*FRAP*	*TPC*	*FRAP*	*TPC*	*FRAP*	*TPC*	*FRAP*
**1**	35 (−1)	38 (−1)	0.05 (−1)	18.70	0.13	20.81	0.29	68.56	0.42	60.94	0.44	59.74	0.34
**2**	35 (−1)	38 (−1)	0.15 (1)	31.77	0.26	93.44	0.93	99.04	1.15	97.34	1.06	91.54	0.83
**3**	35 (−1)	82 (1)	0.05 (−1)	20.18	0.17	60.94	0.35	69.04	0.48	71.04	0.50	61.74	0.34
**4**	35 (−1)	82 (1)	0.15 (1)	37.59	0.33	106.14	0.99	119.44	1.39	126.24	1.15	98.84	0.97
**5**	115 (1)	38 (−1)	0.05 (−1)	19.86	0.16	58.14	0.32	61.34	0.43	63.44	0.45	60.64	0.37
**6**	115 (1)	38 (−1)	0.15 (1)	31.76	0.26	86.84	0.94	99.04	1.40	108.54	1.11	99.94	0.61
**7**	115 (1)	82 (1)	0.05 (−1)	22.68	0.16	75.74	0.41	93.24	0.58	82.04	0.60	62.44	0.43
**8**	115 (1)	82 (1)	0.15 (1)	48.13	0.35	120.14	1.35	163.64	1.50	136.54	1.44	123.84	1.14
**9**	8 (−1.682)	60 (0)	0.1 (0)	5.30	0.08	72.24	0.49	77.24	0.61	76.34	0.55	75.64	0.53
**10**	142 (1.682)	60 (0)	0.1 (0)	30.59	0.25	83.14	0.71	102.54	1.21	100.24	0.96	89.34	0.94
**11**	75 (0)	23 (−1.682)	0.1 (0)	20.31	0.18	69.24	0.49	79.54	0.66	81.54	0.57	73.64	0.51
**12**	75 (0)	97 (1.682)	0.1 (0)	35.61	0.29	95.14	1.08	115.94	1.07	124.34	1.43	104.24	0.96
**13**	75 (0)	60 (0)	0.016 (−1.682)	9.08	0.09	19.38	0.17	59.69	0.21	57.64	0.38	25.35	0.17
**14**	75 (0)	60 (0)	0.184 (1.682)	36.09	0.33	118.64	1.30	138.84	1.34	133.04	1.40	115.64	1.07
**15**	75 (0)	60 (0)	0.1 (0)	27.97	0.25	83.64	1.01	92.54	1.09	106.94	0.94	89.14	0.81
**16**	75 (0)	60 (0)	0.1 (0)	29.24	0.26	94.94	0.79	92.74	0.98	99.24	1.01	85.04	0.78
**17**	75 (0)	60 (0)	0.1 (0)	28.28	0.25	91.34	1.00	94.94	1.16	95.24	0.95	79.44	0.81
**18**	75 (0)	60 (0)	0.1 (0)	27.87	0.25	86.74	0.80	94.14	1.15	103.24	1.24	80.24	0.95

TPC, Total Phenolic Content (mg GAE/g PB); FRAP, Ferric Reducing Antioxidant Power (mmol Fe^2+^/g PB).

**Table 2:** Chemical composition of *Pinus pinaster* bark, expressed as percentage of dry raw material weight (composition by 100 g).

Composition (%)	
**Cellulose^a^**	17.39 ± 0.37
**Hemicellulose**	12.31 ± 0.20
***Xylose***	10.92 ± 0.19
***Arabinose + manose***	1.39 ± 0.01
***Acetyl group***	n.d.
**Lignin**	41.65 ± 0.24
***Klason***	41.05 ± 0.24
***Acid soluble***	0.60 ± 0.00
**Fat**	2.54 ± 0.26
**Protein**	1.64 ± 0.03
**Ash**	0.87 ± 0.00
**Moisture**	8.15 ± 0.02
**Ethanol extractives**	13.20 ± 0.31
**Inorganic substances**	2.56 ± 0.33
**Macro minerals *(Na, K, Ca, Mg, Fe)***	2.54 ± 0.33
**Micro minerals *(Zn, Mn, Cu)***	0.02 ± 0.00

^a^estimated from the glucan content; n.d.: not detected.

**Table 3:** Phenolic compounds identification and quantification in aqueous and hydroethanolic extracts from *Pinus pinaster* bark.

Phenolic Compound (mg/L)	Extracts				
	PB 0%	PB 30%	PB 50%	PB 70%	PB 90%
***Hydroxycinnamic acids***					
caffeic acid	4.2 ± 0.0 ^a^	11.5 ± 0.0 ^b^	13.8 ± 0.6 ^b^	12.0 ± 0.0 ^b^	12.0 ± 0.0 ^b^
ferulic acid	9.7 ± 0.7 ^a^	23.2 ± 2.4 ^b^	24.5 ± 0.1 ^b^	21.2 ± 0.1 ^b^	21.3 ± 0.2 ^b^
cinnamic acid	5.4 ± 0.6 ^a^	29.5 ± 0.3 ^b^	38.1 ± 1.0 ^c^	53.4 ± 2.5 ^d^	47.4 ± 0.2 ^e^
chlorogenic acid	5.8 ± 1.0 ^a^	11.0 ± 0.3 ^b^	15.7 ± 0.1 ^c^	15.5 ± 0.5 ^c^	17.2 ± 0.8 ^d^
*p*-cumaric acid	n.q.	n.q.	n.q.	n.q.	n.q.
***Hydroxybenzoic acids***					
vanillic acid	3.0 ± 1.0 ^a^	8.0 ± 1.0 ^b^	9.5 ± 0.5 ^c^	10.0 ± 0.0 ^c^	10.5 ± 0.5 ^c^
gallic acid	n.q.	n.q.	n.q.	n.q.	n.q.
3,4 dihydroxybenzoic acid	29.7 ± 1.3 ^a^	35.5 ± 1.0 ^b^	36.0 ± 0.3 ^b^	47.4 ± 5.1 ^c^	64.1 ± 1.7 ^d^
ellagic acid	53.6 ± 2.0 ^a^	67.2 ± 0.4 ^b^	120.6 ± 5.1 ^c^	122.6 ± 2.1 ^c^	124.4 ± 16.1 ^c^
***Flavan-3-ols***					
catechin	n.d. ^a^	105.0 ± 1.0 ^b^	133.5 ± 0.9 ^c^	135.5 ± 1.0 ^c^	133.0 ± 2.5 ^c^
gallocatechin	149.3 ± 9.0 ^a^	140.3 ± 7.0 ^a^	n.d. ^b^	n.d. ^b^	n.d. ^b^
epicatechin	n.q.	n.q.	n.q.	n.q.	n.q.
***Flavonoids***					
naringenin	n.d. ^a^	128.0 ± 19.2 ^b^	170.5 ± 18.7 ^c^	249.5 ± 11.0 ^d^	239.6 ± 4.3 ^d^
hesperidin	n.q.	n.q.	n.q.	n.q.	n.q.
quercetin	n.d. ^a^	n.d. ^a^	10.1 ± 0.1 ^b^	8.5 ± 0.5 ^b^	10.9 ± 2.5 ^b^
apigenin	n.d. ^a^	1.9 ± 0.3 ^b^	6.2 ± 0.1 ^c^	12.4 ± 1.2 ^d^	5.1 ± 0.0 ^c^
taxifolin	73.1 ± 11.6 ^a^	166.4 ± 33.9 ^b^	422.9 ± 8.9 ^c^	463.2 ± 6.4 ^d^	463.9 ± 4.4 ^d^
***Stilben***					
resveratrol	3.8 ± 0.1 ^a^	10.9 ± 0.5 ^b^	13.5 ± 0.0 ^c^	18.9 ± 0.3 ^d^	17.5 ± 0.0 ^d^
**Total**	337.6	738.5	1014.9	1171.1	1163.9

Values of phenolic compounds are expressed as concentration (mg/L) mean ± SD of 3 experiments. n.d.: not detected; n.q.: not quantified. Different letters (a–e) show significant differences (*p* < 0.05) for the same compound.

**Table 4:** Antioxidant (DPPH, ABTS and FRAP) and antihyperglycemic (α-Amylase and α-Glucosidase) and antimicrobial activities of aqueous and hydroethanolic extracts from *Pinus pinaster* bark.

Antioxidant Activity						
Extract	PB 0%	PB 30%	PB 50%	PB 70%	PB 90%	Trolox
**DPPH** IC_50_ (µg/mL)	99.96 ± 0.1 ^a^	73.11 ± 0.0 ^b^	49.74 ± 0.1 ^c^	55.04 ± 0.1 ^c^	100.1 ± 0.1 ^a^	10.81 ± 0.1 ^d^
**ABTS** IC_50_ (µg/mL)	106.61 ± 8.0 ^a^	89.18 ± 0.9 ^b^	59.41 ± 2.1 ^c^	65.57 ± 5.0 ^c^	112.1 ± 9.5 ^a^	23.15 ± 4.0 ^d^
**FRAP** (mmol Fe^2+^/g PBE)	101.9 ± 0.3 ^a^	112.4 ± 1.4 ^b^	138.5 ± 4.0 ^c^	122.9 ± 4.6 ^d^	101.3 ± 1.2 ^a^	136.1 ± 1.0 ^c^
**Antihyperglycemic Activity**						
**Extract**	**PB 0%**	**PB 30%**	**PB 50%**	**PB 70%**	**PB 90%**	**Acarbose**
**α-Amylase** IC_50_ (µg/mL)	531.5 ± 5.4 ^a^	536.4 ± 7.1 ^a^	546.3 ± 2.9 ^a^	254.2 ± 9.2 ^b^	300.3 ± 3.9 ^b^	35.42 ± 1.0 ^c^
**α-Glucosidase** IC_50_ (µg/mL)	166.2 ± 1.1 ^a^	132.8 ± 10.8 ^b^	122.7 ± 11.3 ^b^	138.4 ± 7.4 ^b^	162.8 ± 3.7 ^a^	11000 ± 1.0 ^c^
**Antimicrobial Activity** (expressed in zone of inhibition, mm)						
**Extract**	**PB 0%**	**PB 30%**	**PB 50%**	**PB 70%**	**PB 90%**	**LX**
*A. brasiliensis*	n.d.	n.d.	n.d.	n.d.	n.d.	12.5 ± 1.2
*S. cerevisiae*	n.d.	n.d.	n.d.	n.d.	n.d.	39.6 ± 4.2
*C. albicans*	n.d.	n.d.	n.d.	n.d.	n.d.	24.8 ± 0.0
*C. perfringens*	12.7 ± 0.2	13 ± 0.7	13.0 ± 0.2	13.0 ± 0.2	12.7 ± 0.4	18.5 ± 0.8
*B. cereus*	9.6 ± 0.2	10.2 ± 0.0	9.9 ± 0.1	10.8 ± 0.1	10.3 ± 0.2	12.5 ± 2.6
*S. aureus*	9.4 ± 0.2	10.1 ± 0.1	9.7 ± 0.2	10.1 ± 0.3	10.2 ± 0.1	12.5 ± 0.5
*L. monocytogenes*	7.4 ± 0.0	7.8 ± 0.0	7.9 ± 0.6	8.7 ± 0.7	8.3 ± 0.6	9.8 ± 0.3
*E. coli*	n.d.	n.d.	n.d.	n.d.	n.d.	11.4 ± 0.8
*Salmonella* Enteritidis	n.d.	n.d.	n.d.	n.d.	n.d.	12.3 ± 1.2

Values are expressed as mean ± SD of 3–4 experiments. LX: commercial solution of sodium hypochlorite; n.d.: not detected. Different letters show significant differences (p < 0.05) between groups for the same experiment.

## Supplementary Materials

The following are available online at https://www.mdpi.com/2076-3921/9/4/334/s1, Figure S1: Pareto chart. Figure S2: Contour line plots representing the antioxidant activity (FRAP assays) of the tested experimental models. Table S1: Factors and interaction effects of the tested experimental model. Table S2: Quadratic models describing the responses variation of the tested experimental model and their correspondent *R*^2^ coefficients.
