# Supplementary material for: Unravelling the Biological Potential of Pinus pinaster Bark Extracts
Source: Antioxidants (Basel). 2020 Apr 20;9(4):334. doi: 10.3390/antiox9040334 (PMC7222395; doi:10.3390/antiox9040334)
Supplement: Supplementary file 1 [file antioxidants-09-00334-s001.pdf]

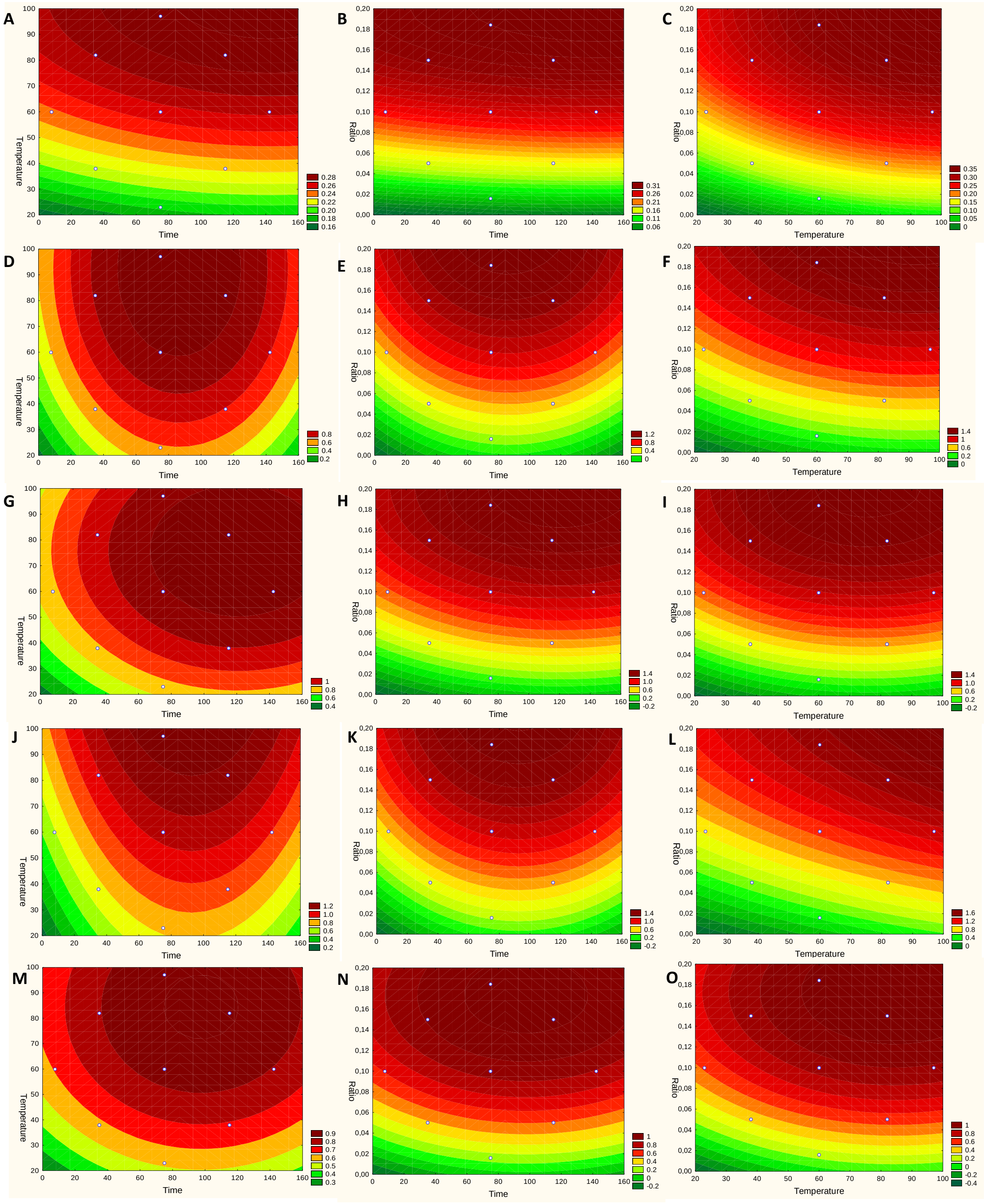

Figure S1. Contour line plots representing the antioxidant activity (FRAP assay) under different conditions of extraction (time, temperature, liquid-solid ratio) of the tested experimental models. EtOH 0% (A,B,C), EtOH 30% (D,E,F), EtOH 50% (G,H,I), EtOH 70% (J,K,L), EtOH 90% (M,N,O).

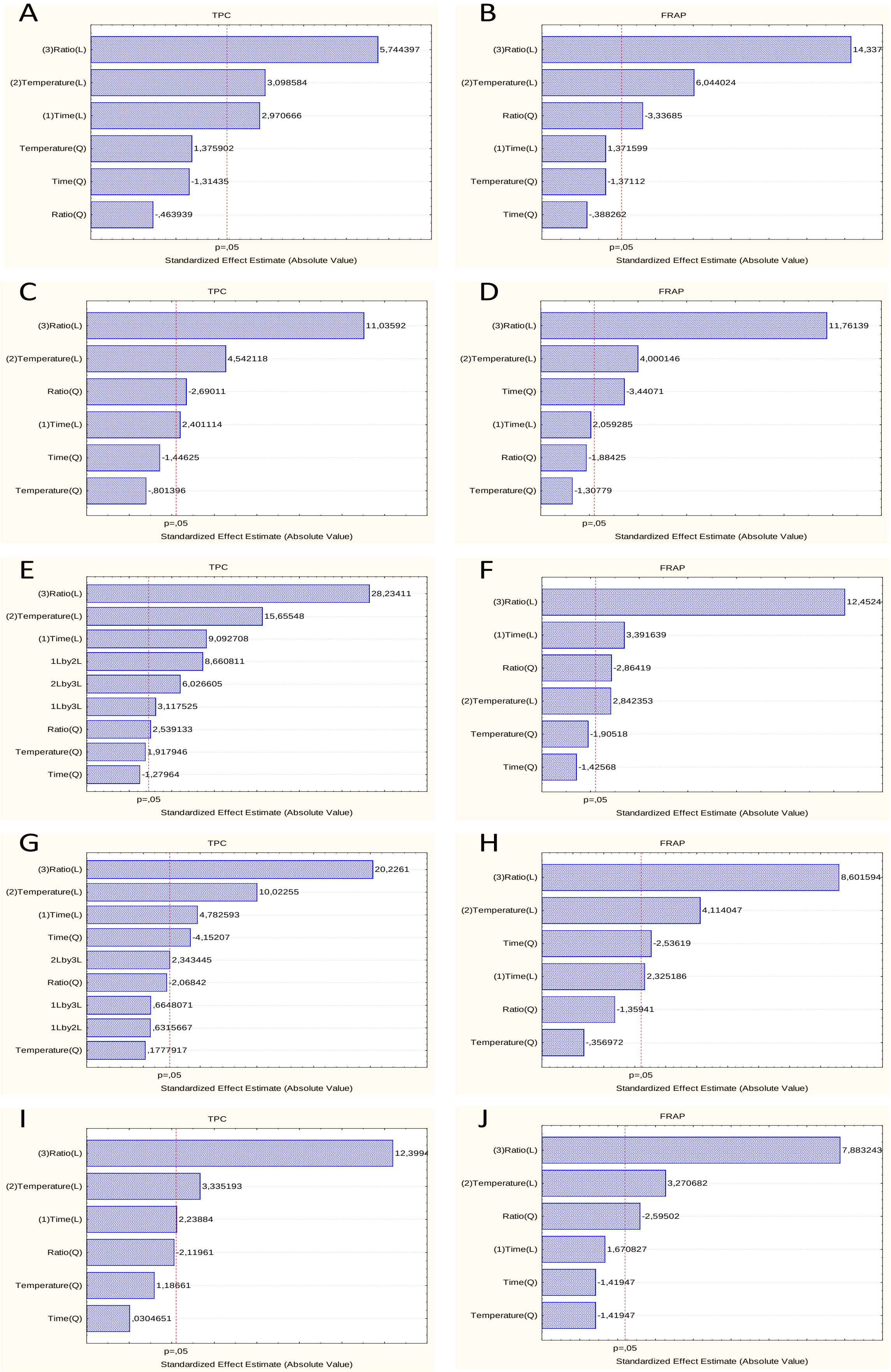

Figure S2. Pareto chart for the effects of time (x_1), temperature (x_2), liquid-solid ratio (x_3), and possible interactions, on the total phenolic content (TPC) (A,C,E,G,I) and FRAP antioxidant activity (B,D,F,H,J) of the tested experimental models. EtOH 0% (A,B), EtOH 30% (C,D), EtOH 50% (E,F), EtOH 70% (G,H), EtOH 90% (I,J). L and Q correspond to the effects at linear and quadratic levels, respectively.

Table S1. Factors and interaction effects of time (x_1), temperature (x_2) and liquid-solid ratio (x_3) on total phenolic content (TPC) and reducing antioxidant activity (FRAP) of the tested experimental model (EtOH 0%, EtOH 30%, EtOH 50%, EtOH 70% and EtOH 90%).

Model	Response	Factor	SS	df	MS	*F-test*	*p-value*
**EtOH 0% (H_2O)**	**TPC**	(1)Time (L)	255.635	1	255.635	8.825	0.013
		Time (Q)	50.042	1	50.042	1.728	0.215
		(2)Temperature (L)	278.124	1	278.125	9.601	0.010
		Temperature (Q)	54.838	1	54.839	1.893	0.196
		(3)Ratio (L)	955.876	1	955.876	32.999	<0.001
		Ratio (Q)	6.235	1	6.235	0.215	0.652
		Error	318.644	11	28.968		
		Total SS	1947.954	17			
	**FRAP**	(1)Time (L)	0.001	1	0.001	1.881	0.198
		Time (Q)	0.001	1	0.000	0.151	0.705
		(2)Temperature (L)	0.014	1	0.013	36.530	<0.001
		Temperature (Q)	0.001	1	0.001	1.880	0.198
		(3)Ratio (L)	0.076	1	0.076	205.571	<0.001
		Ratio (Q)	0.004	1	0.004	11.135	0.006
		Error	0.004	11	0.001		
		Total SS	0.098	17			
**EtOH 30%**	**TPC**	(1)Time (L)	443.91	1	443.910	5.765	0.035
		Time (Q)	161.05	1	161.049	2.092	0.176
		(2)Temperature (L)	1588.50	1	1588.498	20.631	0.001
		Temperature (Q)	49.45	1	49.450	0.642	0.440
		(3)Ratio (L)	9377.50	1	9377.512	121.792	<0.001
		Ratio (Q)	557.20	1	557.199	7.237	0.021
		Error	846.96	11	76.996		
		Total SS	12877.44	17			
	**FRAP**	(1)Time (L)	0.050	1	0.050	4.241	0.064
		Time (Q)	0.141	1	0.141	11.839	0.006
		(2)Temperature (L)	0.190	1	0.190	16.001	0.002
		Temperature (Q)	0.020	1	0.020	1.710	0.218
		(3)Ratio (L)	1.645	1	1.645	138.330	<0.001
		Ratio (Q)	0.042	1	0.0422	3.550	0.086
		Error	0.131	11	0.0122		
		Total SS	2.177	17			
**EtOH 50%**	**TPC**	(1)Time (L)	787.87	1	787.867	82.677	<0.001
		Time (Q)	15.60	1	15.604	1.638	0.237
		(2)Temperature (L)	2335.60	1	2335.602	245.094	<0.001
		Temperature (Q)	35.05	1	35.054	3.679	0.091
		(3)Ratio (L)	7596.51	1	7596.514	797.165	<0.001
		Ratio (Q)	61.44	1	61.438	6.447	0.035
		1L by 2L	714.80	1	714.798	75.010	<0.001

		1L by 3L	92.62	1	92.616	9.719	0.014
		2L by 3L	346.11	1	346.108	36.320	<0.001
		Error	76.24	8	9.529		
		Total SS	12073.57	17			
	**FRAP**	(1)Time (L)	0.160	1	0.160	11.503	0.006
		Time (Q)	0.028	1	0.028	2.033	0.181
		(2)Temperature (L)	0.113	1	0.113	8.079	0.016
		Temperature (Q)	0.051	1	0.051	3.630	0.083
		(3)Ratio (L)	2.159	1	2.159	155.062	<0.001
		Ratio (Q)	0.114	1	0.114	8.207	0.015
		Error	0.153	11	0.014		
		Total SS	2.730	17			
**EtOH 70%**		(1)Time (L)	414.02	1	414.024	22.873	0.001
		Time (Q)	312.05	1	312.053	17.240	0.003
		(2)Temperature (L)	1818.26	1	1818.258	100.452	<0.001
		Temperature (Q)	0.57	1	0.572	0.032	0.863
		(3)Ratio (L)	7404.97	1	7404.968	409.095	<0.001
		Ratio (Q)	77.44	1	77.442	4.278	0.073
		1L by 2L	7.22	1	7.220	0.399	0.545
		1L by 3L	8.00	1	8.000	0.442	0.524
		2L by 3L	99.40	1	99.405	5.492	0.047
		Error	144.81	8	18.101		
		Total SS	10266.68	17			
	**FRAP**	(1)Time (L)	0.113	1	0.113	5.406	0.040
		Time (Q)	0.134	1	0.134	6.432	0.027
		(2)Temperature (L)	0.352	1	0.352	16.925	0.002
		Temperature (Q)	0.003	1	0.003	0.127	0.728
		(3)Ratio (L)	1.540	1	1.540	73.987	<0.001
		Ratio (Q)	0.038	1	0.038	1.848	0.201
		Error	0.229	11	0.021		
		Total SS	2.384	17			
**EtOH 90%**	**TPC**	(1)Time (L)	246.668	1	246.668	5.012	0.047
		Time (Q)	0.046	1	0.046	0.001	0.976
		(2)Temperature (L)	547.405	1	547.405	11.124	0.007
		Temperature (Q)	69.292	1	69.292	1.408	0.260
		(3)Ratio (L)	7566.128	1	7566.128	153.748	<0.001
		Ratio (Q)	221.094	1	221.094	4.493	0.058
		Error	541.326	11	49.211		
		Total SS	9261.411	17			
	**FRAP**	(1)Time (L)	0.042	1	0.042	2.792	0.123
		Time (Q)	0.030	1	0.030	2.015	0.183
		(2)Temperature (L)	0.162	1	0.162	10.698	0.007

Temperature (Q)	0.030	1	0.030	2.015	0.183
(3)Ratio (L)	0.940	1	0.940	62.146	<0.001
Ratio (Q)	0.102	1	0.102	6.734	0.025
Error	0.166	11	0.015		
Total SS	1.434	17			

L and Q correspond to the effects at linear and quadratic levels, respectively.
SS: sum of squares; MS: mean square; df: degrees of freedom.

Table S2. Quadratic models describing the responses variation of total phenolic compounds (TPC) and antioxidant activity (FRAP) in function of independent variables of the tested experimental model (EtOH 0%, EtOH 30%, EtOH 50%, EtOH 70% and EtOH 90%) and their correspondent R^2 coefficients.

Response	Model	Mathematical models [c]	R^2
TPC [a]	EtOH 0%	$27.35 + 4.33x_1 + 4.51x_2 + 8.37x_3$	0.84
	EtOH 30%	$89.06 + 5.70x_1 + 10.78x_2 + 26.20x_3 - 6.64x_3^2$	0.93
	EtOH 50%	$93.55 + 7.60x_1 + 13.08x_2 + 23.58x_3 + 2.20x_3^2 + 9.45x_1x_2 + 3.40x_1x_3 + 6.58x_2x_3$	0.99
	EtOH 70%	$101.26 + 5.51x_1 + 11.54x_2 + 23.29x_3 - 0.10x_1^2 + 3.53x_2x_3$	0.99
	EtOH 90%	$83.37 + 4.25x_1 + 6.33x_2 + 23.54x_3$	0.94
FRAP [b]	EtOH 0%	$0.26 + 0.03x_2 + 0.07x_3 - 0.02x_3^2$	0.96
	EtOH 30%	$0.90 + 0.12x_2 + 0.35x_3 - 0.11x_1^2$	0.94
	EtOH 50%	$1.09 + 0.11x_1 + 0.09x_2 + 0.40x_3 - 0.10x_3^2$	0.94
	EtOH 70%	$1.04 + 0.09x_1 + 0.16x_2 + 0.34x_3 - 0.10x_1^2$	0.90
	EtOH 90%	$0.84 + 0.11x_2 + 0.26x_3 - 0.09x_3^2$	0.88

[a] Total Phenolic Content (mg GAE/g PB); [b] Ferric Reducing Antioxidant Power (mmol Fe^{2+}/g PB); [c] x_1: time; x_2: temperature; x_3: solid-liquid ratio. The equations are expressed in terms of coded values (-1, 0, +1).
